# Isolation and Characterization of *Kingella bonacorsii* sp. nov., A Novel *Kingella* Species Detected in a Stable Periodontitis Subject

**DOI:** 10.3390/pathogens10020240

**Published:** 2021-02-19

**Authors:** Angéline Antezack, Manon Boxberger, Clara Rolland, Virginie Monnet-Corti, Bernard La Scola

**Affiliations:** 1 Faculté des Sciences Médicales et Paramédicales, Ecole de Médecine Dentaire, Aix-Marseille Université, 27 Boulevard Jean Moulin, 13385 Marseille, France; angeline.antezack@univ-amu.fr (A.A.); virginie.corti@univ-amu.fr (V.M.-C.); 2 Assistance Publique-Hôpitaux de Marseille (AP-HM), Hôpital Timone, Service de Parodontologie, 264 rue Saint Pierre, 13385 Marseille, France; 3 Institut de Recherche pour le Développement (IRD), Aix-Marseille Université, Assistance Publique-Hôpitaux de Marseille (AP-HM), MEPHI, 27 Boulevard Jean Moulin, 13005 Marseille, France; manon.boxberger@hotmail.fr (M.B.); rolland.clara@sfr.fr (C.R.); 4 IHU Méditerranée Infection, 19-21 Boulevard Jean Moulin, 13005 Marseille, France

**Keywords:** *Kingella*, dental plaque, periodontitis, culturomics, sp. nov.

## Abstract

Members of the genus *Kingella* are mostly commensals of the oral cavity, but some of them are involved in invasive infections, especially in young children. This study provides new knowledge on the diversity of this genus by describing a novel species of *Kingella* isolated from a dental plaque sample from a 51-year-old man with a history of periodontitis. Morphological and chemotaxonomic characteristic were investigated using different growth conditions, pH and temperature. Cellular fatty acid methyl ester (FAME) analysis was performed by gas chromatography/mass spectrometry (GC/MS). Phylogenetic analysis based on 16S rRNA, orthologous average nucleotide identity (OrthoANI) and digital DNA–DNA hybridization (dDDH) relatedness were also performed. Strain Marseille-Q4569^T^ was found to be a facultative aerobic, nonmotile and non-spore-forming rod-shaped bacterium that grows at 28–41.5 °C (optimum 37 °C), pH 5.5–8.5 (optimum pH 7.5) and 5–15 g/L of NaCl. The major fatty acids were Hexadecanoic acid (32.7%), 11-Octadecenoic acid (26.1 %) and 9-Hexadecenoic acid (21.3 %). Despite high 16S rRNA gene sequence similarity (98.72%) between strain Marseille-Q4569^T^ and *Kingella oralis* strain UB-38^T^, the degree of OrthoANI was at the limit of the cutoff (95.83%), and the degree of dDDH was lower (63.6%) than thresholds used to delineate prokaryotic species. Therefore, it is proposed that strain Marseille-Q4569^T^ represents a novel species of the genus *Kingella*, for which the name *Kingella bonacorsii* sp. nov. is proposed (=CSUR Q4569).

## 1. Introduction

Periodontitis is a chronic inflammatory disease characterized by microbially associated, host-mediated inflammation, which leads to the progressive destruction of the supporting structures of the teeth [1]. Periodontitis can be successfully treated through control of local and systemic risk factors, but stable periodontitis patients must be closely monitored, as they remain at higher risk for recurrent disease. Clinical gingival health after successful treatment of periodontitis was defined by a bleeding on probing < 10% of sites, shallow probing depths of 4 mm or less and no 4 mm sites with bleeding on probing, optimal improvement of other clinical parameters and absence of progressive periodontal destruction [2]. 

The health-associated microbiome is predominantly dominated by *Streptococcus*, *Actinomyces*, *Rothia*, *Lautropia*, *Bergeyella*, *Kingella* and *Granulicatella* species [3]. The genus *Kingella* (N.L. fem. dim. n. *Kingella*, named after Elizabeth O. King, an American bacteriologist) was first introduced into the literature by Henriksen and Bøvre [4] in 1976 and currently counts six species. Although *Kingella* species are mostly commensals of the oral cavity, they can cause human diseases such as *Kingella kingae*, which is described as a prime cause of invasive infections, including septic arthritis, osteomyelitis and spondylodiscitis in young children [5]. Grevich et al. [6] reported that juvenile idiopathic arthritis patients had significantly more gingival inflammation, and plaque microbiota analysis revealed bacteria belonging to genera *Kingella* elevated. By contrast, other authors showed that periodontal inflammation, in case of gingivitis or periodontitis, was characterized by a reduction of *Kingella* species [7,8]. 

In the present study, we used the rapid, cost-effective routine identification by matrix-assisted laser desorption ionization time-of-flight (MALDI-TOF) mass spectrometry (MS) for the identification of an unknown strain, which was isolated from a dental plaque sample from a 51-year-old man with clinical gingival health following successful treatment of periodontitis. Strain Marseille-Q4569^T^ was then described using data from next-generation sequencing data, morphological examinations and biochemical characteristics. We propose to establish for this strain the species name *Kingella bonacorsii* sp. nov.

## 2. Results

### 2.1. Strain Isolation and Phenotypic Characteristics

**Figure 1:**
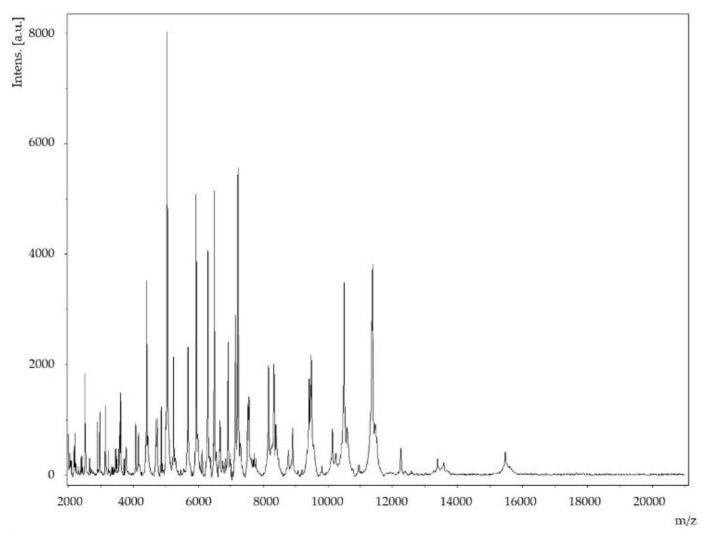
MALDI-TOF MS reference mass spectrum for strain Marseille-Q4569^T^. Spectra from 12 individual colonies were compared, and a reference spectrum was generated.

**Figure 2:**
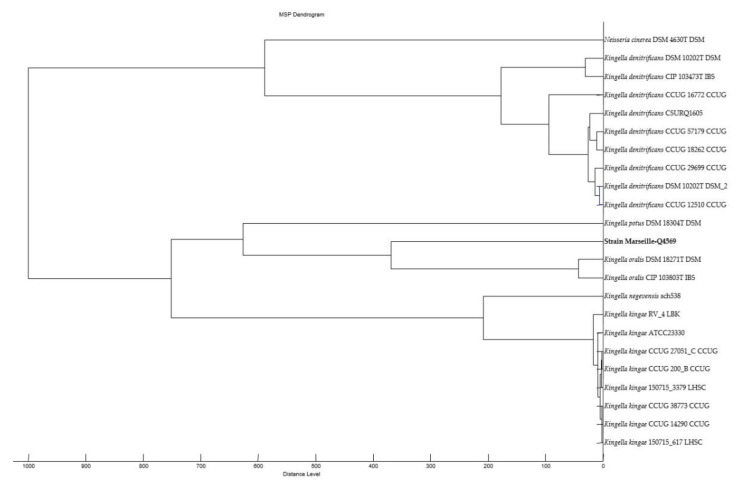
MALDI-TOF MS dendrogram highlighting the position of strain Marseille-Q4569 within the *Kingella* genus.

Strain Marseille-Q4569^T^ was isolated from a dental plaque sample from a 51-year-old man with clinical gingival health following successful treatment of periodontitis and living in Marseille, France. Strain Marseille-Q4569^T^ could not be identified by MALDI-TOF MS, as the score was lower than 1.8 against the MEPHI (Microbes, Evolution, Phylogénie, Infection) database. The reference spectrum of strain Marseille-Q4569^T^ (Figure 1) did not belong to any known *Kingella* species (Figure 2). Growth was observed on 5% sheep blood-enriched Columbia agar (BioMérieux, Marcy l’Etoile, France) at 37 °C after 24 h of incubation under aerobic atmosphere. Growth was also achieved in anaerobic (AnaeroGen Compact; Oxoid, Thermo Scientific, Dardilly, France) and microaerophilic atmospheres (campyGEN; Oxoid, Thermo Scientific, Dardilly, France). The bacterial cells tolerated a temperature ranging from 28 °C to 41.5 °C (optimum 37 °C) and a pH of 5.5 to 8.5 (optimum pH 7.5). It was determined that the NaCL content of the strain was between 5 and 15 g/L, the optimum being 5 g/L.

**Figure 3:**
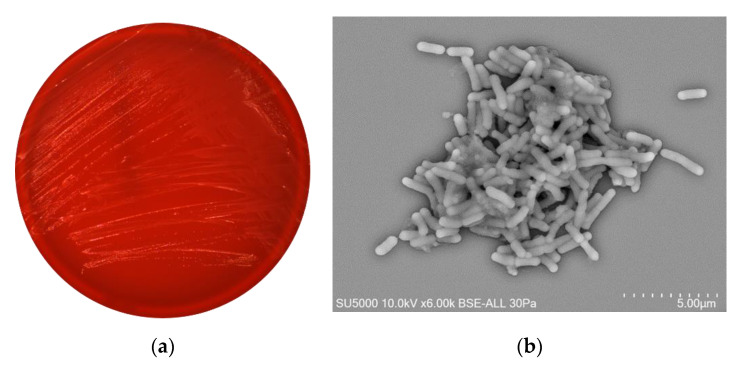
Morphological characteristics of strain Marseille-Q4569^T^. (**a**) Microcolonies of strain Marseille-Q4569^T^ on 5% sheep blood-enriched Columbia agar (BioMérieux, Marcy l’Etoile, France) incubated at 37 °C for 2 days. (**b**) Micrograph electron microscopy of strain Marseille-Q4569^T^ using a SU5000 FE-SEM (Hitachi High-Tech, HHT, Tokyo, Japan). Scales and acquisition are shown on the figure.

Microcolonies on 5% sheep blood-enriched Columbia agar (BioMérieux, Marcy l’Etoile, France) incubated at 37 °C for 2 days were translucent, convex, with circular regular edges and with a mean diameter of 1.0 mm. The bacterial cells were Gram-negative, straight rods about 0.4 to 0.7μm wide and 1.3 to 2.6μm long with round tips (Figure 3).

The sporulation test was negative. Strain Marseille Q4569^T^ showed oxidase activity but was negative for catalase. According to the API 50CH strip, no acid production was observed. Using an API ZYM strip, positive reactions were observed for alkaline phosphatase, C_4_ esterase, C_8_ esterase lipase, leucine arylamidase, acid phosphatase and naphtol-AS-BI-phosphohydrolase. Negative reactions were recorded for C_14_ lipase, valine arylamidase, cystine arylamidase, trypsin, α-chymotrypsin, α-galactosidase, β-galactosidase, β-glucuronidase, α-glucosidase, β-glucosidase, N-acetyl-β-glucosaminidase, α-mannosidase and α-fucosidase (Table 1).

The major fatty acids were Hexadecanoic acid (32.7%), 11-Octadecenoic acid (26.1 %) and 9-Hexadecenoic acid (21.3 %). Several branched and 3-hydroxy structures were also described (Table 2).

### 2.2. Genome Sequencing Information and Genome Properties

**Figure 4:**
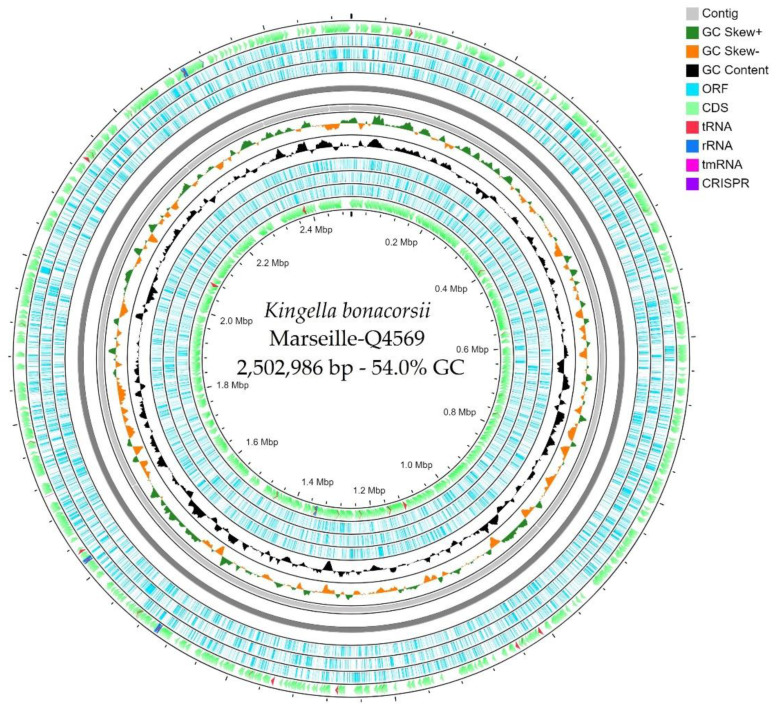
A circular map generated using the CGView Server^BETA^ [14] showing a complete view of the genome of strain Marseille-Q4569^T^.

The genome size of strain Marseille-Q4569^T^ was 2,502,986 bp long with a 54.0% G+C content. The genome assembly was achieved on 9 contigs with a mean coverage of 36.0. It was deposited into GenBank under the accession number JAEHNZ000000000. Of the 2698 predicted genes, 2556 were protein-coding genes, 71 were RNAs (4 5S rRNA, 4 16S rRNA, 4 23S rRNA, 55 tRNAs and 4 ncRNA), and 71 were pseudo genes. Genes with putative function (by clusters of orthologous groups, COGs) were 1353 for *Kingella bonacorsii* strain Marseille-Q4569^T^ (Table 3). Finally, 1177 genes (43.6%) were annotated as hypothetical proteins for strain Marseille-Q4569^T^. A circular map showing a complete view of the genome of strain Marseille-Q4569^T^ is shown in Figure 4.

### 2.3. Comparison with Closely Related Bacterial Strains

**Figure 5:**
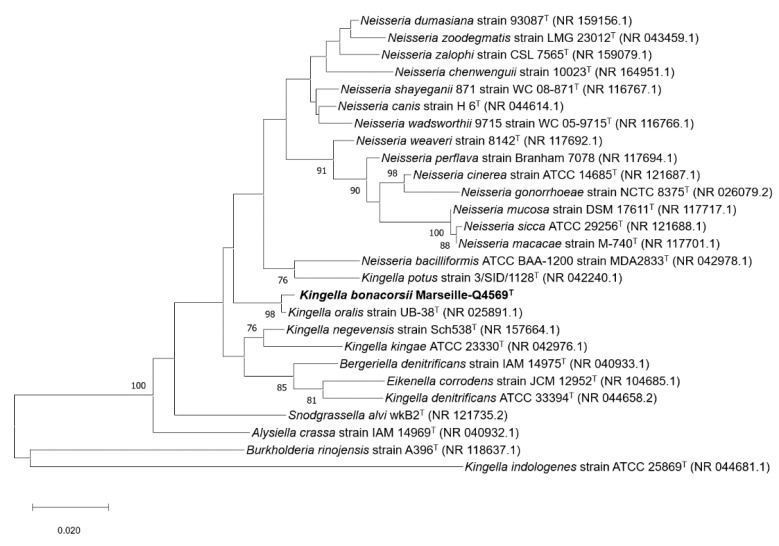
Maximum likelihood tree based on the comparison of 16S rRNA gene sequences showing the phylogenetic relationships of strain Marseille-Q4569^T^ and other closely related species. Bootstrap values (expressed as percentages of 1,000 replications) are displayed at the nodes. Only bootstrap values of 70% or greater are shown. Type strains are indicated with superscript T. GenBank accession numbers of 16S rRNA are indicated in parentheses. Sequences were aligned using MUSCLE (MUltiple Sequence Comparison by Log- Expectation) with default parameters, phylogenetic inference were obtained using the maximum likelihood method and the MEGA X software [15]. The bootstrap values obtained by repeating the analysis 1000 times to generate a majority consensus tree are shown at the nodes. There was a total of 1360 positions in the final dataset.

The 16S rDNA-based similarity analysis of strain Marseille-Q4569^T^ against GenBank yielded highest nucleotide sequence similarities of 98.72 % sequence identity with *K. oralis* strain UB-38^T^ (GenBank accession no. NR_025891.1). The 16S rRNA gene sequence was deposited into GenBank under the accession number MW367549. The phylogenetic tree highlighting the position of the strain relative to other closely related species is shown in Figure 5. 

The genome of strain Marseille-Q4569^T^ was compared to the available genomes of nine closely related bacterial strains. The genome size of our strain (2.50 Mb) was larger than that of *N. cinerea*, *K. kingae*, *B. denitrificans*, *N. shayeganii*, *K. oralis*, *N. zalophi* and *N. chenwenguii* (1.83 Mb, 2.00 Mb, 2.32 Mb, 2.35 Mb, 2.40 Mb, 2.42 Mb, 2.49 Mb, respectively), but smaller than that of *S. alvi* (2.52 Mb) and *N. canis* (2.56 Mb). The G+C content of our strain (54.0%) was equal to or higher than that of all strains compared, with the exception of *K. oralis* (54.3%), *B. denitrificans* (55.8%) and *N. shayeganii* (56.4%). When performing digital DNA–DNA hybridization (dDDH) analysis, strain Marseille-Q4569^T^ exhibited values ranging from 63.6 (60.7–66.4)% with *K. oralis* to 22.2 (19.9–24.6)% with *N. canis* (Table 4).

**Figure 6:**
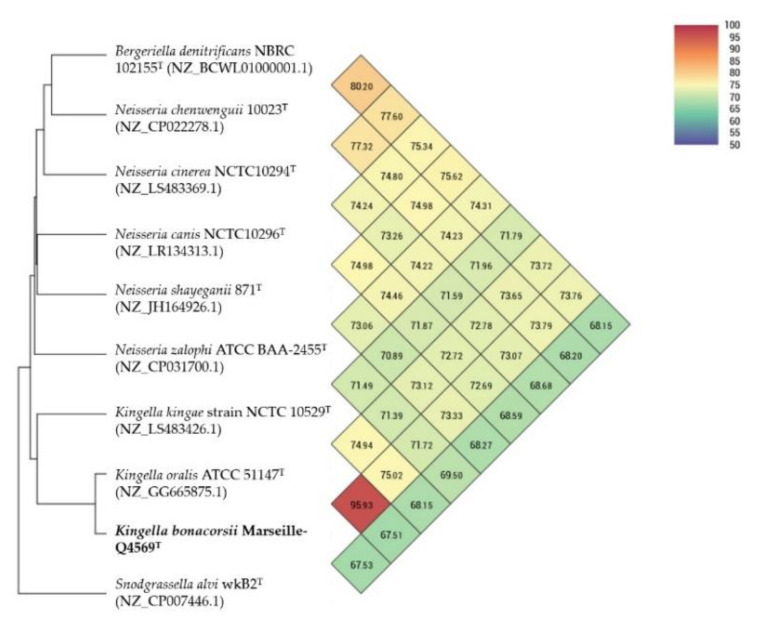
Heatmap generated with orthologous average nucleotide identity (OrthoANI) values calculated using the OAT software between of strain Marseille Q4569^T^ and nine other closely related species.

These dDDH values are below the 70% threshold used to delineate prokaryotic species, confirming that strain Marseille-Q4569^T^ represents a new specie [10]. Furthermore, orthologous average nucleotide identity (OrthoANI) analysis among closely related species shows that the highest percentage value was shared at 95.83% between *K. bonacorsii* and *K. oralis*, while the lowest was 67.51% between *S. alvi* and *K. oralis* (Figure 6).

**Figure 7:**
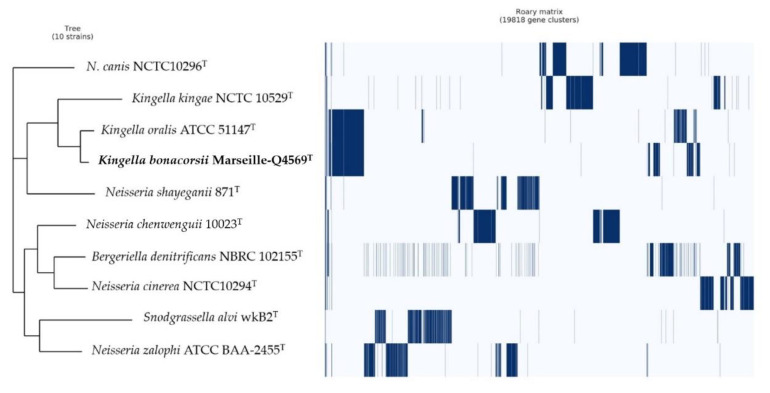
Pangenome analysis of strain Marseille-Q4569^T^ whole-genome sequences. A maximum likelihood tree was constructed from the accessory genome elements (left). The presence (blue) and the absence (white) of accessory elements of the genome are presented on the right.

Pangenome analysis of strain Marseille-Q4569^T^ showed a total of 19,818 clusters genes distributed as follows: (Core genes = 5), (Soft core genes = 0), (Shell genes = 1790) and (Cloud genes = 18,023), respectively (Figure 7).

Finally, despite a high 16S rRNA gene sequence similarity with *Kingella oralis* strain UB-38^T^ (98.72 %) [17] and a degree of orthologous average nucleotide identity (OrthoANI) at the limit of the cutoff (95.83%) [18], the degree of DNA–DNA relatedness (dDDH) below the thresholds used to delineate prokaryotic species [16] associated with nonidentification by MALDI-TOF MS confirmed that strain Marseille-Q4569^T^ represents a new species.

## 3. Discussion

*Kingella bonacorsii* (Bo.na.cor’si.i N.L. gen. masc. n. named after Stephane Bonacorsi, a Professor of clinical microbiology in France and main national expert of *Kingella* sp. bone and joint infections). Cells were Gram-staining-negative, facultative aerobic, rod-shaped, about 0.4 to 0.7μm wide and 1.3 to 2.6μm long with round ends. Colonies on Columbia agar with 5% sheep blood (Biomérieux, Marcy l’Etoile, France) were translucent and circular. The temperature range for growth was 28 to 41.5 °C (optimum 37 °C). The cells grew at pH 5.5–8.5 (optimum pH 7.5) and in the presence of 5–15 g/L NaCl. Strain Marseille Q4569^T^ showed oxidase activity but were negative for catalase. Positive reactions were observed for alkaline phosphatase, C_4_ esterase, C_8_ esterase lipase, leucine arylamidase, acid phosphatase and naphtol-AS-BI-phosphohydrolase. Negative reactions were recorded for C_14_ lipase, valine arylamidase, cystine arylamidase, trypsin, α-chymotrypsin, α-galactosidase, β-galactosidase, β-glucuronidase, α-glucosidase, β-glucosidase, N-acetyl-β-glucosamidase, α-mannosidase and α-fucosidase. The major fatty acids were Hexadecanoic acid (32.7 %), 11-Octadecenoic acid (26.1 %) and 9-Hexadecenoic acid (21.3 %). Several branched and 3-hydroxy structures were also described. DNA G+C content of the type strain was 54.0 %. 

The type strain, Marseille-Q4569^T^ (CSUR Q4569), was isolated from a dental plaque sample from a successfully treated stable periodontitis patient. The discovery of this new species, alone, does not allow us to associate its presence with the patient’s periodontal condition. In this aim, additional studies exploring the prevalence of our strain *Kingella bonacorsii* Marseille-Q4569^T^ according to the presence or absence of periodontal diseases will be of great interest. The sequence data of the 16S rRNA gene of Marseille-Q4569^T^ and the whole genome have been deposited in the GenBank database under accession numbers MW367549 and JAEHNZ000000000, respectively.

## 4. Materials and Methods

### 4.1. Strain Isolation and Phenotypic Tests

A dental plaque sample was taken from a 51-year-old man living in Marseille, France, and presenting a clinical gingival health following a successful treatment of periodontitis. The patient provided a signed informed consent, and the study was approved by the Comité de Protection des Personnes (C.P.P.) Sud-Ouest et Outre-Mer 1 (n ID RCB: 2020-A01234-35—CPP 1-20-075 ID 9806). Briefly, the sampling area was first isolated by cotton rolls and gently air-dried for 5 s to remove any saliva present. Supragingival plaque was collected using a sterile curette and placed into a 1.5 mL Eppendorf tube containing 1.0 mL of Aé-Ana transport medium (Culture-Top, Eurobio scientific, Les Ulis, France). After vortexing for 30 s, a 10-fold dilution series of the sample was prepared in phosphate-buffered saline 1×. Columbia sheep blood agar plates (Biomerieux, Marcy l’Etoile, France) were inoculated with 50μL of each 10^−4^ to 10^−8^ diluted plaque suspension. After 48 h of incubation under aerobic atmosphere at 37 °C, culture plates were inspected using a magnifying glass, and any microcolonies or colonies showing satellitism were passaged onto a fresh Columbia sheep blood agar plate. MALDI-TOF MS protein analysis was performed with a Microflex LT mass spectrometer (Bruker Daltonics, Bremen, Germany; external mass spectrometer calibration accuracy ± 300 ppm), as previously reported [19]. Briefly, each isolate colony was deposited on a 96-polished steel MALDI target and then coated with 1 μL of a matrix solution containing α-cyano-4-hydroxycinnamic acid diluted into 500 μL of acetonitrile, 250 μL of 10% trifluoroacetic acid and 250 μL of HPLCgrade water. The matrix sample was then crystallized by air-drying at room temperature, as previously described [20]. Two spots were systematically created for each colony, and each isolate was characterized by at least 12 spots. The obtained spectra were imported into the BioTyper-RTC^TM^ version 3.0 software (Bruker Daltonics GmbH, Bremen, Germany) and analyzed by standard pattern matching (with default parameter settings) against the constantly updated MEPHI database (https://www.mediterranee-infection.com/acces-ressources/base-de-donnees/urms-data-base/, (accessed on 4 January 2021). Interpretation of the scores was carried out as previously reported [19]. A log score ≥ 2.0 was considered an identification with high confidence, according to the manufacturer (Bruker Daltonics GmbH). One purified strain, designated Marseille-Q4569^T^, and deposited in the Collection de Souches de l’Unité des Rickettsies under accession number Q4569, could not be identified by MALDI-TOF MS.

Gram staining was carried out using the standard Gram stain, and morphological characteristics were observed using a field emission scanning electron microscope (SU5000 FE-SEM, Hitachi High-Tech, HHT, Tokyo, Japan) using cultures grown on Columbia agar with 5% sheep blood media (Biomérieux, Marcy l’Étoile, France) at 37 °C for 24 h under aerobic condition. A colony was collected from agar and immersed into a 2.5% glutaraldehyde fixative solution. The slide was gently washed in water, air-dried and examined with a SU5000 FE-SEM operated at 10.0 kV.

Subculture of strain Marseille-Q4569^T^ was attempted at a wide range of temperatures (25, 28, 31.5, 37, 41.5 and 56 °C) on Columbia Agar with a 5% sheep blood medium, and under different pH (5.5, 6.5, 7.5 and 8.5) and salinity (5, 10 and 15 g/L) conditions on a Columbia Agar base (bioMérieux, Marcy l’Étoile, France). Growth of the strain was also tested under anaerobic (AnaeroGen Compact; Oxoid, Thermo Scientific, Dardilly, France) and microaerophilic (campyGEN; Oxoid, Thermo Scientific, Dardilly, France) conditions at 37 °C for 48 h. API ZYM and API 50 CH kits (bioMérieux, Marcy l’Étoile, France) were used to perform biochemical analysis, according to the manufacturer’s instructions. To assess whether our strain was able to form spores, a heat shock at 80 °C during 10 min was operated. Oxidase (MASTDISCS^®^ ID, Mast Group Ltd., Bootle, Merseyside, UK) and catalase (bioMérieux, Marcy l’Étoile, France) assays were also performed. Finally, fatty acid methyl ester (FAME) was operated by gas chromatography/mass spectrometry (GC/MS) as previously reported [21,22]. Two samples were prepared with approximately 3 mg of bacterial biomass per tube harvested from several culture plates. Then, fatty acid methyl esters were separated using an Elite 5-MS column and monitored by mass spectrometry (Clarus 500-SQ 8 S, Perkin Elmer, Courtaboeuf, Villebon sur Yvette, France). Spectral database search was performed using MS Search 2.0 operated with the Standard Reference Database 1A (NIST, Gaithersburg, MD, USA) and the FAMEs mass spectral database (Wiley, Chichester, UK). 

### 4.2. Extraction and Genome Sequencing

Genomic DNA was extracted with the EZ1 biorobot (Qiagen, Courtaboeuf, Les Ulis, France) with the EZ1 DNA tissue kit and then sequenced on the MiSeq technology (Illumina, San Diego, CA, USA) using the the Nextera Mate Pair sample prep kit and Nextera XT Paired end (Illumina, San Diego, CA, USA), as previously described [23]. In order to improve the genome sequence, the Oxford Nanopore approach was operated on 1D genomic DNA sequencing for the MinIon device using SQK-LSK109 kit. A library was then constructed from 1 µg genomic DNA without fragmentation and end repair. Adapters were ligated to both ends of genomic DNA. After purification on AMPure XP beads (Beckman Coulter Inc, Fullerton, CA, USA), a Qubit assay quantified the library with the high sensitivity kit (Life technologies, Carlsbad, CA, USA). In total, 1159 active pores were detected for sequencing, and the workflow WIMP was chosen for live bioinformatic analysis. After 1.5 h run time and end-of-life of the flowcell, 82,460 reads as raw data were generated. 

### 4.3. Assembly and Annotation of Genome Sequence

A pipeline incorporating different software (Velvet [24], Spades [25], Soap Denovo [26]) and trimmed data (MiSeq and Trimmomatic [27] software) or untrimmed data (only MiSeq software) was used to perform the assembly. The reduction of assembly gaps was obtained with GapCloser. Scaffolds <800 bp and scaffolds with a depth value <25% of the mean depth were removed. The best assembly was selected using different criteria (number of scaffolds, N50, number of N).

The prediction in the open reading frame (ORF) was performed using Prokka (Galaxy v 1.14.5) with default settings [28]. Deviations in the sequencing regions predicted by ORFs were excluded. BlastP was used to predict the bacterial proteome (E value of 1e03, coverage of 70% and percent identity of 30) according to the orthological group (COG) database. In the absence of a match, the search for BlastP in the database [29] was extended with an E value of 1e03, coverage of 70% and percent identity of 30. On the other hand, if the length of the sequence was less than 80 amino acids (aa), an E value of 1e05 was used. The rRNA and tRNA genes were retrieved using Prokka (Galaxy v 1.14.5) [30,31]. In addition, CGView Server^BETA^ [14] was used to generate a circular map displaying a complete view of the genome of strain Marseille-Q4569^T^.

### 4.4. Phylogenetic Analysis and Genome Comparison

The 16S rRNA gene sequence of strain Marseille-Q4569^T^ was obtained and compared to the closest related species retrieved using the National Center for Biotechnology Information, Basic Local Alignment Search Tool (NCBI BLAST; https://blast.ncbi.nlm.nih.gov/Blast.cgi, (accessed on 30 December 2020)) and then submitted to the GenBank database. Phylogenetic analyses were performed using MEGA X software [23], with genetic distances determined according to the Kimura two-parameter model [24] and phylogenies reconstructed with the maximum-likelihood method. The topology of the phylogenetic tree was conducted using the bootstrap method with 1000 repetitions. All positions containing gaps and missing data were eliminated from the dataset (complete deletion option). To estimate the mean level of nucleotide sequence similarity at the genome level between strain Marseille-Q4569^T^ and closely related species, the digital DNA–DNA hybridization (dDDH) as well as the orthologous average nucleotide identity (OrthoANI v 0.93.1) parameters were calculated using the OAT [18] and GGDC (Genome-to-Genome Distance Calculator v 2.1) [16] software programs, respectively. The Pangenome distribution of strain Marseille-Q4569^T^ and other closely related species was assessed using the Roary software (Galaxy v 3.13.0) [32].

## 5. Conclusions

The present study characterized a novel species of *Kingella*, based on both morphology and phylogeny. As some members of the *Kingella* genera are clearly identified as key pathogens in several inflammatory diseases, additional studies exploring the virulence potential of our strain *Kingella bonacorsii* Marseille-Q4569^T^ will be of great interest. 

## Figures and Tables

**Table 1:** Phenotypic and biochemical characterization of strain Marseille-Q4569^T^ compared with 6 other *Kingella* species. API ZYM and API 50 CH test kits (bioMérieux) were used for the characterization of strain Marseille-Q4569^T^. +, Positive; −, negative; ND, no data available.

Characteristics	Strain Marseille-Q4569^T^	*Kingella negevensis* Sch538^T^ [9]	*Kingella kingae* ATCC 23330^T^ [4,9]	*Kingella indologenes* NCTC 10717^T^ [10]	*Kingella denitrificans* NCTC 10995^T^ [10,11,12]	*Kingella oralis* UB-38^T^ [11]	*Kingella potus* 3/SID/1128^T^
Rods or coccobacilli	+	+	+	+	+	+	+
Pigment	−	−	−	−	−	−	+
Catalase	−	−	−	−	−	−	−
Glucose	−	−	+	+	+	+	−
Maltose	−	−	+	+	−	−	−
Fructose	−	−	−	+	−	−	−
Alkaline phosphatase	+	+	ND	ND	ND	+	−
C_4_ esterase	+	−	ND	ND	ND	ND	ND
C_8_ esterase lipase	+	−	ND	ND	ND	ND	ND
C_14_ lipase	−	−	ND	ND	ND	ND	ND
Leucine arylamidase	+	+	ND	ND	ND	ND	ND
Valine arylamidase	−	−	ND	ND	ND	ND	ND
Cystine arylamidase	−	−	ND	ND	ND	ND	ND
Trypsin	−	−	ND	ND	ND	ND	ND
α-Chymotrypsin	−	−	ND	ND	ND	ND	ND
Acid phosphatase	+	+	ND	ND	ND	ND	ND
Naphthol-AS-BI-Phosphohydrolase	+	−	ND	ND	ND	ND	ND
α-Galactosidase	−	−	ND	ND	ND	ND	ND
β-Galactosidase	−	−	−	−	−	−	−
β-Glucuronidase	−	−	ND	ND	ND	ND	−
α-Glucosidase	−	−	ND	ND	ND	ND	−
β-Glucosidase	−	−	ND	ND	ND	ND	ND
N-Acetyl-β-Glucosaminidase	−	−	ND	ND	ND	ND	ND
α-Mannosidase	−	−	ND	ND	ND	ND	ND
α-Fucosidase	−	−	ND	ND	ND	ND	ND

**Table 2:** Cellular fatty acid compositions of strain Marseille-Q4569^T^ and other *Kingella* species. All values are given as a percentage of the total fatty acids. Strains: 1, Marseille-Q4569^T^; 2, *Kingella kingae* ATCC 23330^T^ [9]; 3, *Kingella potus* 3/SID/1128^T^ [13]; 4, *Kingella negevensis* Sch538^T^ [9]. −, Not detected; TR, trace amounts (<1%); ND, do data available.

Fatty Acid	1	2	3	4
C_10:0_ 3-OH	TR	ND	3.0	ND
C11:0	ND	ND	2.0	ND
C_12:0_	1.9	5.6	5.2	22.5
C_12:0_ 3-OH	2.3	2.3	3.6	6.5
C_14:0_	4.9	40.0	2.7	37.7
C_14:0_ 3-OH	TR	1.1	3.0	4.3
C_14:1_	TR	1.5	ND	−
C_14:1n5_	−	TR	ND	5.7
C_15:0_	TR	1.1	2.1	TR
C_16:0_	32.7	10.8	34.8	19.5
C_16:1n7_	21.3	25.5	14.4	18.3
C_17:0_	TR	ND	0.9	ND
C_18:0_	1.7	2.2	1.0	7.1
C_18:1n7_	26.1	6.5	18.5	2.2
C_18:1n9_	3.4	1.4	2.4	7.7
C_18:2n6_	4.2	TR	ND	3.7

**Table 3:** Number of genes associated with the clusters of orthologous group (COG) functional categories of strain Marseille-Q4569^T^.

Code	Strain Marseille-Q4569^T^	Description
[J]	139	Translation, ribosomal structure and biogenesis
[A]	1	RNA processing and modification
[K]	60	Transcription
[L]	109	Replication, recombination and repair
[B]	0	Chromatin structure and dynamics
[D]	26	Cell cycle control, cell division, chromosome partitioning
[Y]	0	Nuclear structure
[V]	18	Defense mechanisms
[T]	22	Signal transduction mechanisms
[M]	99	Cell wall/membrane/envelope biogenesis
[N]	1	Cell motility
[Z]	0	Cytoskeleton
[W]	0	Extracellular structures
[U]	26	Intracellular trafficking, secretion and vesicular transport
[O]	54	Posttranslational modification, protein turnover, chaperones
[X]	0	Mobilome: prophages, transposons
[C]	86	Energy production and conversion
[G]	47	Carbohydrate transport and metabolism
[E]	107	Amino acid transport and metabolism
[F]	43	Nucleotide transport and metabolism
[H]	65	Coenzyme transport and metabolism
[I]	42	Lipid transport and metabolism
[P]	67	Inorganic ion transport and metabolism
[Q]	11	Secondary metabolites biosynthesis, transport and catabolism
[R]	165	General function prediction only
[S]	165	Function unknown

**Table 4:** Numerical DNA–DNA hybridization values (%) obtained by comparison between strain Marseille-Q4569^T^ and other closely related species using Genome-to-Genome Distance Calculator 2 (GGDC 2) [16]. Confidence intervals indicate the inherent uncertainty in estimating DNA–DNA hybridization values from intergenomic distances, based on models derived from empirical test data sets.

Species	1	2	3	4	5	6	7	8	9	10
1 *Kingella bonacorsii* Marseille-Q4569^T^	100.00	63.6 [60.7–66.4]	33.3 [30.9–35.8]	24.0 [21.7–26.5]	23.2 [20.9–25.6]	22.9 [20.6–25.4]	22.5 [20.2–24.9]	22.4 [20.1–24.9]	22.3 [20–24.7]	22.2 [19.9–24.6]
2 *Kingella oralis* ATCC 51147^T^ (NZ_GG665875.1)		100.00	30.8 [28.4–33.3]	23.6 [21.3–26.1]	22.4 [20.1–24.8]	22.2 [19.9–24.6]	21.5 [19.2–23.9]	21.7 [19.5–24.1]	21.9 [19.6–24.3]	21.8 [19.5–24.2]
3 *Snodgrassella alvi* wkB2^T^ (NZ_CP007446.1)			100.00	25.7 [23.4–28.2]	23.1 [20.8–25.6]	25.1 [22.8–27.6]	22.9 [20.6–25.4]	26.8 [24.4–29.3]	25.2 [22.8–27.6]	22.8 [20.5–25.2]
4 *Neisseria cinerea* NCTC10294^T^ (NZ_LS483369.1)				100.00	23.0 [20.8–25.5]	24.0 [21.6–26.4]	24.1 [21.8–26.6]	23.7 [21.4–26.1]	23.3 [21–25.8]	23.2 [20.9–25.7]
5 *Neisseria zalophi* ATCC BAA-2455^T^ (NZ_CP031700.1)					100.00	22.4 [20.1–24.8]	21.9 [19.7–24.4]	24.8 [22.5–27.3]	23.9 [21.6–26.3]	22.8 [20.5–25.3]
6 *Neisseria chenwenguii* 10023^T^ (NZ_CP022278.1)						100.00	20.9 [22.8–27.6]	22.9 [20.7–25.4]	22.2 [19.9–24.6]	23.2 [21–25.7]
7 *Bergeriella denitrificans* NBRC 102155^T^ (NZ_BCWL01000001.1)							100.00	22.4 [20.2–24.9]	22.4 [20.1–24.8]	23.7 [21.4–26.2]
8 *Neisseria shayeganii* 871^T^ (NZ_JH164926.1)								100.00	23.5 [21.2–26]	26.7 [24.4–29.2]
9. *Kingella kingae* strain NCTC 10529^T^ (NZ_LS483426.1)									100.00	22.3 [20–24.7]
*10 Neisseria canis* NCTC10296^T^ (NZ_LR134313.1)										100.00

## Data Availability

The data presented in this study is contained within the article.
